# TET1 Protects the Lungs from Diesel Exhaust Particle-Induced Inflammation and Abnormal Function

**DOI:** 10.21203/rs.3.rs-9860246/v1

**Published:** 2026-06-19

**Authors:** Stephanie N. Henson, Anthony P. Brown, Sweeney P. Elston, Evan Holmes, Hong Ji

**Affiliations:** University of California, Davis; University of California, Davis; University of California, Davis; University of California, Davis; University of California, Davis

**Keywords:** TET1, particulate matter, lung inflammation, aryl hydrocarbon receptor, neutrophil

## Abstract

**Rationale:**

Diesel exhaust particles (DEP) are a major contributor to air pollution-associated asthma exacerbations, promoting oxidative stress, airway inflammation, and Th17-skewed immune responses. Although epigenetic mechanisms are increasingly recognized as key modulators of environmental lung disease, the role of the DNA demethylation enzyme TET1 in DEP-induced airway dysfunction remains poorly defined. We investigated the contribution of TET1 to epithelial and airway responses to DEP using complementary human and murine models.

**Methods:**

TET1 was silenced in human bronchial epithelial cells (HBECs) followed by DEP exposure, and transcriptomic alterations and cytokine production were assessed. In parallel, Tet1 heterozygous (Tet1^+/−^) and wild-type littermates were subjected to repeated intratracheal DEP exposure to evaluate airway hyperresponsiveness, lung inflammation, immune cell recruitment, and cytokine expression. Mechanistically targeted interventions supporting TET activity, inhibiting aryl hydrocarbon receptor (AhR) signaling, or limiting mitochondrial oxidative stress were evaluated *in vitro* and *in vivo*.

**Results:**

Loss of TET1 markedly amplified DEP-induced transcriptional responses in HBECs, characterized by enhanced pro-inflammatory signaling and suppression of AhR-dependent xenobiotic detoxification genes. *In vivo*, Tet1^+/−^ mice exhibited increased airway hyperresponsiveness, neutrophilic inflammation, and elevated expression of neutrophil chemokine and Th17-associated cytokines following DEP exposure. Notably, pharmacologic AhR inhibition and reduction of oxidative stress attenuated inflammatory cytokine expression *in vitro* and partially mitigated DEP-induced airway inflammation and hyperresponsiveness *in vivo*.

**Conclusions:**

These findings suggest that epigenetic modulation of TET1-dependent pathways may represent a novel strategy to reduce susceptibility to pollution-induced airway disease. Collectively, our data identify TET1 as a central epigenetic regulator integrating detoxification and inflammatory programs in the airway epithelium during environmental exposure.

## Introduction

Air pollution is a critical risk factor in the development and exacerbation of lung diseases, especially asthma^1^. Environmental exposures, including air pollution, can induce changes in the epigenome, which have been linked to the increased risk of lung disease^1–3^. However, the molecular mechanisms directly involved in air pollution-promoted asthma are not fully understood. In 2022, 26.8 million Americans had asthma, and 11.3 million people had at least one asthma attack (American Lung Association report). Children younger than 5 and females had higher risks of asthma attack than other groups (67.9% and 45.3%, respectively, in 2022). The annual cost of asthma is estimated at $3,266 per person per year, more than half of which is related to prescription medication costs^4^. While the standard-of-care treatments effectively control clinical asthma symptoms, they cannot prevent the natural course of the disease and are associated with significant side effects with long-term usage^5–7^. Therefore, it is necessary to examine the molecular mechanisms involved in asthma to develop new and curative therapeutics.

TET1 (Tet Methylcytosine Dioxygenase 1) is a member of ten-eleven translocation methylcytosine dioxygenase family, which initiates DNA demethylation, and is an important regulator of epigenetic modifications. Specifically, TET1 can catalyze the hydroxylation of 5-methyl-Cytosine into 5-hydroxyl-methyl-Cytosine, 5-formylcytosine (5fC), and 5-carboxycytosine (5caC). However, DNA methylation is not the only epigenetic modification in which TET1 is involved. TET1 also interacts with histone modifiers and transcription factors to play a role in many key biological processes and responses to environmental exposures (reviewed in^8^). In our previous study^9^, we found that lung inflammation, airway mucus secretion, and airway hyper-responsiveness (AHR) were markedly enhanced by *Tet1* knockout in a mouse model of house dust mite (HDM)-induced allergic airway inflammation, suggesting that TET1 could play a role in protecting the lung from inflammation and the development of asthma-related symptoms in response to allergens.

Diesel exhaust particles (DEP) are a major particulate component of traffic-related air pollution (TRAP), which consists of a carbonaceous core, a group of organic components (e.g. halogenated aromatic hydrocarbons, polycyclic aromatic hydrocarbons, and redox-active quinones), and adsorbed materials (e.g. soot, metals and allergens)^1^. DEP is commonly used in research studies as a surrogate for TRAP. Previous studies have established that exposure to TRAP or DEP promotes airway inflammation and oxidative stress^1^. Additionally, DEP exacerbates lung function impairment and disease severity in both asthma patients and asthmatic animal models^1,10,11^. In particular, a Th17 immune response resulting from DEP exposure increases the risk of severe and persistent asthma^10,11^. This Th17 response is characterized by neutrophilia and regulated by proinflammatory cytokines such as IL17a and IL17f. In a co-exposure study, Brandt et al. showed that DEP enhanced Th2 and Th17 cytokines production, particularly IL-33, IL-5, IL-13, and IL-17A, and intensified airway hyper-responsiveness (AHR) and airway mucus secretion in HDM-induced asthmatic mouse model^11^. Meanwhile, they found that DEP alone only induced lung inflammation, without AHR, in this model.

We previously showed that TET1 may dysregulate genes in the aryl hydrocarbon receptor (AhR) signaling pathway in an allergic inflammation model^9^. The Aryl hydrocarbon receptor (AhR) pathway plays a key role in lung inflammation and oxidative stress. AhR can be activated by environmental pollutants like benzopyrene in DEP. Upon activation, AhR translocates to the nucleus, dimerizes with ARNT or interacts with other transcription factors, and induces the transcription of genes encoding enzymes and cytokines involved in inflammation. Additionally, the Nrf2-mediated pathway is activated to combat reactive oxidative species (ROS), upregulating detoxification enzymes like Glutathione S-transferases (GSTs)^12^. However, excessive ROS can lead to protein misfolding, activating the unfolded protein response and inflammatory pathways which further promote lung inflammation and remodeling^13,14^.

Recently, several studies, including ours^12–14^, suggest that DNA methylation plays an important role in DEP-induced lung inflammation both in human and in animal models. In our previous study^15^, we found that DEP significantly altered genome-wide 5mC and 5hmC patterns in human bronchial epithelial cells (HBECs), which were accompanied by dynamic changes in the expression of *DNMT1*, *DNMT3A* and *TET1*. The CpG sites with altered DNA methylation were located in genes and pathways related to oxidative stress responses, epithelial function, and immune cell responses. We previously showed that DEP exposure altered *TET1* expression in HBECs in a time- and dose-dependent manner^16^. Specifically, low-dose DEP exposure (5 μg/cm^2^) transiently increased *TET1* expression at 1 hour post-exposure, with levels returning to baseline by 4 and 24 hours. In contrast, higher doses (10 and 20 μg/cm^2^) produced a sustained decrease in *TET1* expression at 24 hours post-exposure. However, it remains unclear whether these changes reflect an adaptive protective response or a pathological loss of epigenetic regulation that contributes to lung inflammation. Therefore, in this study, we utilize well-established models of DEP exposure and propose to test whether TET1 deficiency exacerbates DEP-induced lung inflammation. We also aim to determine the role of TET1 in oxidative stress responses, neutrophil recruitment and Th17 responses and the contribution of this role in DEP-induced inflammation. To this end, we analyzed RNA-seq data from an *in vitro* cell culture model of DEP exposure and determined how TET1 loss affected expression in major pathways involved in oxidative stress response. We also assessed lung inflammation and function in Tet1 deficient mice and wildtype littermates and determined the impact of pharmacologically promoting TET protein activity and targeting oxidative stress response pathways on lung inflammation.

## Results

### TET1 deficiency in vitro leads to overlapping transcriptomic changes with those induced by DEP.

**Figure 1:**
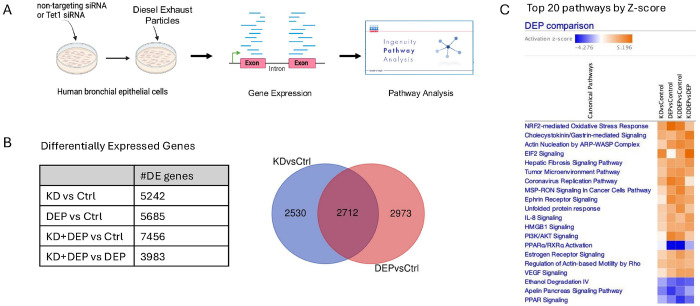
TET1 knockdown and DEP challenge significantly changed the transcriptome of human bronchial epithelial cells (HBECs). (A) Experimental design. (B) Differentially expressed (DE) genes. The table shows the number of DE genes in each comparison group: Tet1 knockdown (KD) vs. Control, DEP vs. Control, KD+DEP vs. Control, and KD+DEP vs. DEP. The Venn diagram illustrates overlap between DE genes in KD vs. Control and DEP vs. Control comparisons. (C) Pathway analysis using QIAGEN Ingenuity Pathway Analysis. Heatmap displays the top 20 canonical pathways ranked by activation Z-score shared between experimental comparisons (KDvsControl, DEPvsControl, KD+DEPvsDEP, KD+DEPvsControl). Positive Z-score (Orange) indicates activation; Negative Z-score (Blue) indicates inhibition.

Our previous studies showed that DEP exposure altered TET1 expression in HBECs in a time- and dose-dependent manner^16^. However, the specific gene networks and molecular pathways through which TET1 mediates the transcriptional response to DEP exposure remain poorly defined. To understand how TET1 regulates gene expression to respond to DEP, we compared transcriptomic changes induced by TET1 knockdown (~ 70% knockdown, as published in^17^) and DEP exposure (10 μg/cm^2^) using RNA-seq (study design in Fig. 1A). TET1 KD and DEP exposure each produced significant transcriptomic changes in HBECs (KD vs Ctrl: 5242 DEGs; DEP vs Ctrl: 5685 DEGs) (Fig. 1B). The combined KD and DEP condition yielded the largest transcriptional change relative to control (KD + DEP vs Ctrl: 7456 DEGs), suggesting a compounded or synergistic effect between these two conditions. The direct comparison between KD + DEP and DEP (3,983 DEGs) highlights the extent to which TET1 loss modifies the transcriptional response to DEP exposure. The Venn diagram comparing KD vs Ctrl and DEP vs Ctrl (Fig. 1B) shows that 2,712 genes were commonly differentially expressed in both conditions, while 2,530 genes were uniquely altered by TET1 knockdown and 2,973 were uniquely affected by DEP exposure. This suggests that while there were a core set of genes influenced by both conditions, each condition also uniquely affected gene expression.

Pathway analysis using the Ingenuity Pathway Analysis (IPA) tool revealed the top 20 enriched pathways with the highest Z-scores across all four comparisons, indicating predicted activation or inhibition (Fig. 1C). Many of these pathways are well-documented in the context of diesel particle exposure, including the Nrf2 oxidative stress response and cytokine signaling pathways involving IL-17 and IL-8. Notably, TET1 knockdown alone appears to impact these pathways even in the absence of DEP, suggesting that TET1 plays a broader role in regulating inflammatory and oxidative stress responses. This finding indicates that the loss of TET1 may predispose cells to an altered baseline state that could influence how they respond to environmental stressors like DEP.

### TET1 deficiency in vitro dysregulates transcriptomic responses to DEP.

**Figure 2:**
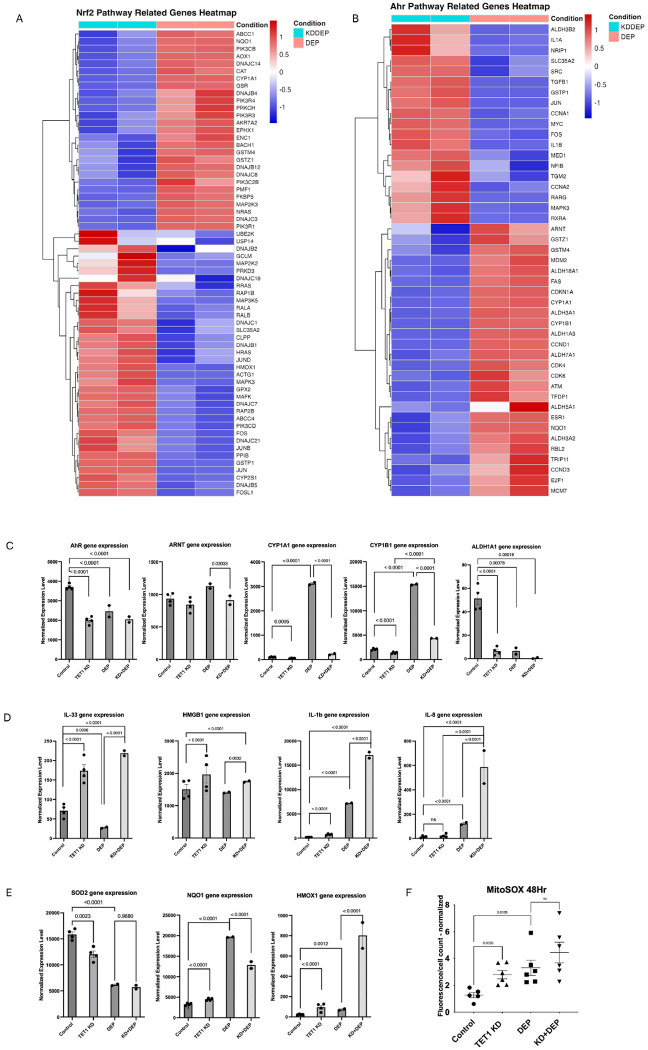
TET1 deficiency enhanced pro-inflammatory responses to DEP and disrupts AhR signaling and oxidative stress response. Heatmap of selected differentially expressed genes in the (A) Nrf2 oxidative stress pathway and (B) AhR signaling pathway. Comparison of KD+DEP (teal; n=2) versus DEP (pink; n=2) conditions. (C) Gene expression of pro-inflammatory genes in HBECs from RNA-seq. Data shown as mean ± SEM; p-adj values from differential expression analysis (D) Gene expression of genes in the AhR signaling pathway. Data shown as mean ± SEM; p-adj values from differential expression analysis of RNA-seq data. (E) Gene expression of antioxidant enzyme genes. Data shown as mean ± SEM; p-adj values from differential expression analysis. (F) Mitochondrial superoxide levels measured by MitoSOX Red staining assay at 48 hours post DEP treatment, normalized by cell counts. Welch’s t tests were applied. Data shown as mean ± SEM.

Given the prominence of immune-regulatory, xenobiotic, and stress-response pathways in our analyses, we further examined Nrf2 oxidative stress and aryl hydrocarbon receptor (AhR) signaling in the context of DEP exposure and TET1 deficiency. IPA predicted activation of the NRF2-mediated oxidative stress response (Z-score = 2.197) and the AhR signaling pathway (Z-score = 0.354) in KD + DEP versus DEP, prompting a closer examination of genes within these pathways. Heatmaps of NRF2- and AhR-pathway genes revealed broad transcriptional dysregulation in KD + DEP HBECs compared with DEP, including changes in NRF2 targets (e.g., HMOX1, NQO1, GST genes) and canonical AhR components/targets (ARNT, CYP1A1, CYP1B1, ALDHs) (Fig. 2A–B). Gene-level differential expression analysis revealed that TET1 knockdown modified key components of these pathways. Several key genes required for AhR function were downregulated, including ARNT, an essential AhR cofactor needed for nuclear translocation, as well as detoxification-related downstream genes such as CYP1A1, CYP1B1, and multiple aldehyde dehydrogenases (ALDHs) (**Figure C**). Conversely, among the upregulated genes were pro-inflammatory cytokines IL-1β and IL-1α (Fig. 2B–C). These findings suggest that TET1 loss disrupts normal AhR signaling, potentially impairing the cell’s ability to respond to xenobiotic stress while simultaneously promoting a heightened inflammatory state.

Due to the well-established links between AhR signaling and immune regulation, we further examined whether inflammatory mediators were similarly dysregulated. Several pro-inflammatory mediators were significantly dysregulated across experimental conditions (Fig. 2D). Notably, IL33 and HMGB1, two alarmins that are rapidly released in response to cellular stress or damage, were significantly increased following TET1 knockdown. Conversely, in the DEP group *HMGB1* expression was not elevated and IL-33 was decreased. Since alarmins are early response genes, the 24-hour time point used in this experiment may not optimally capture this response. Importantly, both IL33 and HMGB1 were significantly elevated in the KD + DEP group compared to control. This pattern suggests that loss of TET1 may prolong alarmin expression beyond its normal resolution phase or otherwise disrupt the temporal regulation of cytokine signaling. Furthermore, IL1B, a potent pro-inflammatory cytokine, was significantly increased following TET1 knockdown alone. Consistent with previous studies, IL1B expression was also significantly upregulated in response to DEP exposure^3,10,18,19^. This effect was further amplified in the KD + DEP condition, indicating a potential synergistic interaction between TET1 loss and DEP exposure in promoting IL-1β expression (Fig. 2D). A similar pattern was observed for IL8, a key chemokine involved in neutrophil recruitment. DEP exposure increased IL8 expression, and TET1 knockdown in the context of DEP exposure further enhanced this response (Fig. 2D).

Detoxification and oxidative-stress pathways are central to limiting reactive oxygen species (ROS). Therefore, we next examined redox-related genes (Fig. 2E) and mitochondrial superoxide production (Fig. 2F). SOD2, which encodes mitochondrial superoxide dismutase (MnSOD), is a key antioxidant enzyme in the mitochondrial matrix^20^. We observed significantly decreased SOD2 expression in response to TET1 knockdown and DEP exposure (Fig. 2E). NQO1, another Nrf2-regulated antioxidant gene, was significantly increased by TET1 knockdown and further increased by DEP. While it was increased by DEP in TET1-KD cells, KD + DEP had significantly lower expression than DEP alone, suggesting that the effect of TET1 on NQO1 expression depends on DEP exposure. In contrast, HMOX1, a widely used biomarker of oxidative stress and Nrf2 pathway activation^30,31^, was significantly elevated in both the TET1 KD and DEP groups and was further increased in the KD + DEP group, consistent with heightened oxidative burden in TET1-deficient cells (Fig. 2E). Functionally, mitochondrial superoxide measured by MitoSOX was increased when TET1 was knocked down, and this increase persisted in the presence of DEP (Fig. 2F). Collectively, these findings support a role for TET1 in regulating oxidative-stress defenses, particularly those linked to mitochondrial redox balance, and indicate that its loss can exacerbate DEP-associated cellular stress by weakening detoxification/antioxidant functions while concurrently promoting inflammation.

### Loss of TET1 exacerbates DEP-induced airway hyperresponsiveness and lung inflammation in mice.

**Figure 3:**
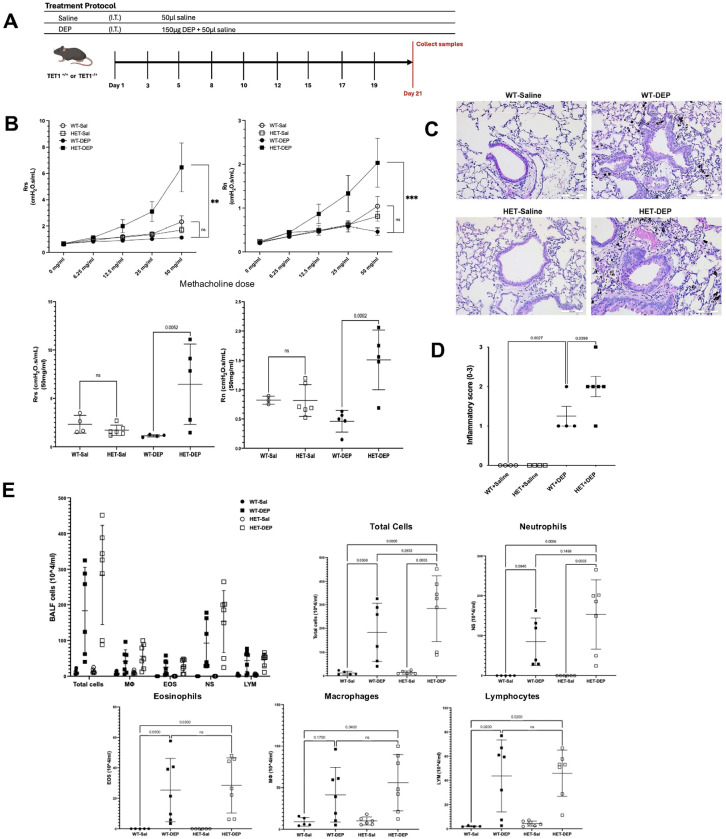
TET1 deficiency in mice exacerbates lung inflammation and abnormal lung function in response to DEP. (A) Experimental design. TET1 heterozygous mutant (HET) and wild-type littermates (WT) mice were administered with intratracheal (IT) instillations of either saline or DEP (150 mg) 3 times per week for 3 weeks (9 IT instillations in total). (B) Airway hyper-responsiveness (AHR) in murine model of DEP-induced lung inflammation. (top) The changes in respiratory system resistance (Rrs) and airway resistance (Rn) in different dosages of methacholine. *p < 0.05, **p < 0.01, ***p < 0.001; (bottom) Rrs and Rn with 50mg/ml methacholine. One-way ANOVA with Šídák's multiple comparisons tests were applied. The outliners were excluded, which were identified by Grubbs test. (C) Lung histology (H&E staining). A representative of all mice in the experiment is shown. A representative of all mice in the experiment is shown. (D) Quantification of lung inflammation, scored by the presence of inflammatory cells (see methods). (E) The concentrations of inflammatory cells in BALF. (Top row) Summary of all cell types, Total Cells, Neutrophils (NS); (Bottom row) Eosinophils (EOS), Macrophages (Mφ), Lymphocytes (LYM); One-way ANOVA with Tukey's post hoc tests were applied. Data shown as mean ± SEM. Animal numbers: WT-Sal, n=3–5; WT-DEP, n=6–7; HET-Sal, n=5–6; HET-DEP, n=5–7.

To investigate the effects of TET1 loss *in vivo*, we utilized a mouse model with Tet1 heterozygous (Tet1^+/−^, HET mice) and littermate wild-type (WT) mice. We employed Tet1 heterozygous (Tet1^+/−^) mice rather than complete knockout mice to model a more physiologically relevant state of partial TET1 deficiency, which may better reflect the dynamic, exposure-dependent changes in TET1 expression observed in human epithelial cells^15,16^. In addition, TET1 plays an important role in embryonic development, and complete knockout mice exhibit reduced litter sizes and other developmental defects^21–23^, further supporting the use of a heterozygous model. These mice underwent a previously established 3-week intratracheal (IT) exposure protocol for diesel particle-induced lung inflammation^10,11^. In this model, the doses of DEP reliably induce neutrophilic lung inflammation and Th17 responses without causing AHR in wild-type mice^10,11^, providing a sensitive window to detect exacerbated responses in Tet1-deficient mice. Forty-eight hours after the final exposure, we assessed lung pathology, airway hyper-responsiveness (AHR), immune cell counts and cytokine levels in bronchial alveolar lavage fluid (BALF), and lung mRNA expression (Fig. 3A). Consistent with this model, DEP treatment did not significantly impact AHR in WT mice, however, we observed significantly increased AHR in HET mice when treated with DEP (Fig. 3B). As expected, DEP exposure led to significant lung inflammation in WT mice, evidenced by significant histological changes and increased BALF total cells and neutrophils (Fig. 3C–E). However, these responses were even more pronounced in TET1-deficient mice, indicating that TET1 plays a protective role in modulating the inflammatory response. The increased inflammation in HET mice was characterized by heightened immune cell infiltration, epithelial cell hyperplasia, and thickening of the basement membrane, all of which contributed to an elevated inflammation score (Fig. 3D). Both WT and HET mice exhibited an increase in total immune cells and neutrophils following diesel particle exposure, consistent with a Th17-driven inflammatory response. However, the increase in immune cell infiltration was even more pronounced in HET mice; neutrophils were significantly increased in HET-DEP vs. HET-Sal, but not in WT-DEP vs. WT-Sal (Fig. 3E). These findings suggest that TET1 deficiency exacerbates DEP-induced lung inflammation, further supporting its role in regulating airway immune responses and tissue remodeling.

### Molecular characteristics of mouse lungs and BALF associated with AHR and cellularity.

**Figure 4:**
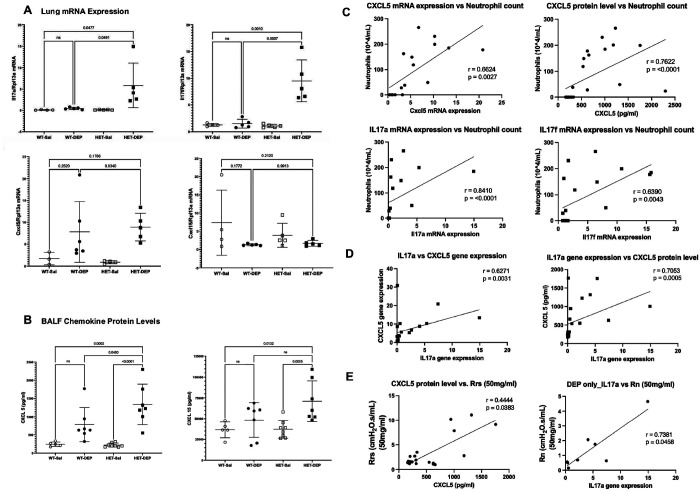
Molecular characteristics of mouse lungs and bronchoalveolar lavage fluid (BALF) associated with airway hyperresponsiveness and cellularity. (A) Relative mRNA expression of inflammatory cytokines (IL17a, IL17f, CXCL5, CXCL15) in lung tissue measured by qPCR, normalized to Rpl13a gene. (B) Chemokine protein levels (CXCL5, CXCL15) in BALF measured by ELISA. One-way ANOVA with Tukey's post hoc tests were applied. The outliners identified by Grubbs test were excluded. (C) Spearman correlation analyses were performed between (top) BALF neutrophil counts and BALF chemokine concentrations (CXCL5 and CXCL15) and (bottom) lung cytokine gene expression (IL17a and IL17f). (D) Spearman correlation analyses were performed between airway hyperresponsiveness measurements and (left) BALF chemokine concentration (CXCL5) and (right) lung cytokine gene expression (IL17a). Only DEP groups (WT+DEP and HET+DEP) were included in this analysis. For both C and D, points show individual observations; a linear regression line is displayed for visualization of the overall trend in each plot. Animal numbers: WT-Sal, n=3–5; WT-DEP, n=6–7; HET-Sal, n=5–6; HET-DEP, n=5–7.

Examining lung gene expression together with BALF chemokine protein levels provided additional insight into the inflammatory phenotype associated with DEP exposure and TET1 deficiency. We quantified the mRNA of Th17-associated cytokines (Il17a and Il17f) that promote neutrophil recruitment and activation and neutrophil-recruiting chemokines (Cxcl5 and Cxcl15) in lung tissue (Fig. 4A), and measured BALF CXCL5 and CXCL15 protein (Fig. 4B). We observed significantly elevated IL17a and IL7f mRNA in HET-DEP mice compared to WT-DEP mice (Fig. 4A). While there were no significant changes in CXCL5 or CXCL15 gene expression, the protein levels of both chemokines were significantly increased in the HET + DEP group compared to WT + Sal (Fig. 4B). Additionally, CXCL5 protein levels were significantly higher in HET + DEP group compared to WT + DEP (Fig. 4B), suggesting that TET1 deficiency may have a more significant impact on CXCL5 than CXCL15. Additional genes (including Il13, Il33, Il1β and Il6) and BALF proteins (IL-1β and TNF-α) were measured (**Supplemental Fig. 1A–B**), although no significant changes were observed.

To further characterize this model, we preformed Spearman correlation analyses between the measured cytokine/chemokines and observed inflammatory and airway phenotypes, including neutrophilia and airway hyperresponsiveness (**Supplementary Table 1**). Specifically, BALF neutrophil counts were strongly correlated with BALF CXCL5 and CXCL15 protein concentrations (Fig. 4C and **Supplemental Table 1**; CXCL5: r = 0.7622, p < 0.0001; CXCL15: r = 0.6215, p = 0.0009), supporting a link between these chemokines and neutrophilic airway inflammation in this model. Because neutrophilic inflammation is often associated with Th17-related cytokine responses, we next evaluated Il17a and Il17f expression and their relationship to inflammatory cellularity. BALF neutrophils correlated strongly with lung Il17a expression (r = 0.841, p < 0.0001) and Il17f expression (r = 0.639, p = 0.0043) (Fig. 4C and **Supplementary Table 1**), consistent with an IL-17–linked neutrophilic signature in DEP-exposed HET mice. Il17a expression was also positively correlated with lung Cxcl5 gene expression (r = 0.6271, p = 0.0031) and with BALF CXCL5 protein levels (r = 0.7053, p = 0.0005) (Fig. 4D), consistent with the idea that these cytokines/chemokines are characteristic markers in a Th17 immune response. Additionally, Rrs at the 50 mg/ml methacholine dose was positively correlated with BALF CXCL5 protein (r = 0.4444, p = 0.0383) (Fig. 4E and **Supplementary Table 1**), supporting an association between CXCL5-linked inflammation and airway resistance. Notably, CXCL5 seems to have stronger correlation to functional readouts (neutrophilia and AHR) than CXCL15 and may be a better marker for this model (Fig. 4C–E and **Supplementary Table 1**). Furthermore, as demonstrated in Fig. 3, there were key similarities in the changes seen in Il17a expression and airway hyperresponsiveness, specifically in the DEP treated animals. Therefore, we assessed the correlation between IL17a expression and airway hyperresponsiveness when restricting the analysis to DEP groups (WT + DEP and HET + DEP). In a DEP-only subset analysis, Il17a mRNA levels were also associated with Rn at 50 mg/ml (r = 0.7381, p = 0.0458) (Fig. 4E and **Supplementary Table 1**), further connecting Il17 to airway physiology under DEP exposure. Collectively, these data support the hypothesis that TET1 regulates airway inflammatory responses by modulating cytokine signaling and immune cell recruitment promoting neutrophil and Th17 responses.

### Targeting TET1 and TET1-regulated pathways reduces DEP-induced inflammatory responses in vitro and in vivo and mitigates airway hyperresponsiveness in Tet1^+/−^ mice.

AhR signaling and oxidative stress responses emerged as key drivers of DEP-induced inflammatory gene expression in both our *in vitro* (Fig. 2) and *in vivo* (**Supplemental Fig. 2**) models, where TET1 deficiency further exacerbated dysregulation of these AhR and Nrf2 pathways. Therefore, we sought to investigate potential therapeutic molecules that could target these pathways to alleviate this inflammatory response. We focused on three compounds: Dimethyl 2-oxoglutarate, CH223191, and SS-31. Dimethyl 2-oxoglutarate (DMAKG), a cell-permeable precursor of α-ketoglutarate, was used to increase intracellular α-ketoglutarate, the essential co-substrate of TET dioxygenases that potentially increase TET1 catalytic activity^24,25^. CH223191, a ligand-selective AhR antagonist, was used to counteract AhR activation, which is commonly induced by DEP exposure and transcriptionally modulate downstream xenobiotic response and proinflammatory cytokine production^26,27^. Finally, SS-31 (Elamipretide), a synthetic peptide known to improve mitochondrial function and reduce ROS^20^, was included to address the oxidative stress associated with DEP exposure.

**Figure 5:**
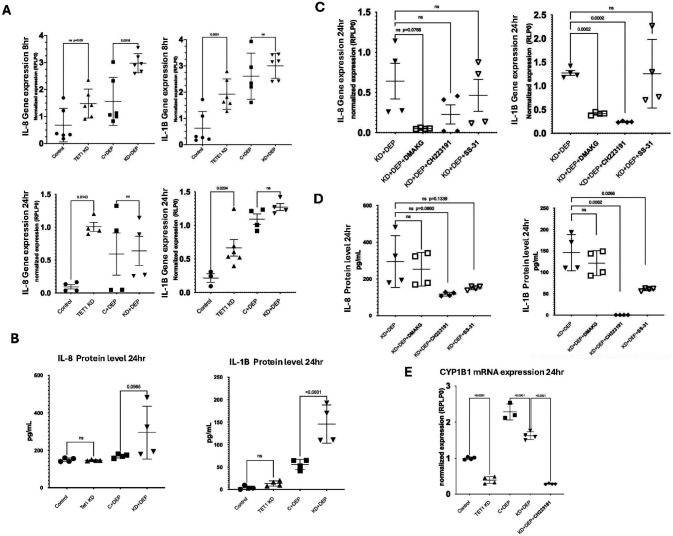
Targeting TET1 and TET1-regulated pathways in HBECs reduced proinflammatory cytokines following DEP exposure. (A, C, E) Relative mRNA expression of proinflammatory cytokines (IL8, IL1b and CYP1B1) were measured by qPCR. (B and D) Cytokine protein levels measured in cell culture supernatants by ELISA. (A, B) Effect of TET1 KD and DEP exposure on cytokine levels. One-way ANOVA with Šídák's multiple comparisons test were applied. (C, D) Effect of treatments (DMAKG, CH223191, SS-31) on cytokine levels compared to KD+DEP group. Welch’s t tests were applied.

In HBECs, TET1 knockdown significantly increased inflammatory cytokine IL1B mRNA expression at both 8hr and 24hr and increased IL8 mRNA at 24hr, with a trend toward increased IL8 already evident at 8hr (p = 0.08) (Fig. 5A). Additionally, IL8 and IL1B are increased at both timepoints in the KD + DEP group compared the C + DEP group, although the increase is only significant for IL8 at 8hr. The difference in KD + DEP versus C + DEP is more prevalent and significant in the protein levels of IL8 and IL1B (Fig. 5B), as well as consistent with our observations from RNA-seq analysis (Fig. 2D). We next evaluated whether our proposed treatments (DMAKG, CH223191, SS-31) could mitigate the heightened KD + DEP inflammatory state. Compared to the untreated groups, treatments with DMAKG (400 μM), CH223191 (10 μM), or SS-31 (100 nM) reduced IL8 and IL1B gene expression at 24hr (Fig. 5C) and decreased corresponding secreted cytokine protein levels to varying degrees (Fig. 5D). While DMAKG effectively reduced IL1B gene expression and showed a trend of reducing IL8, this treatment did not sufficiently reduce protein levels. CH223191 significantly reduced IL1B gene expression and protein levels (**Figure C and D**), and SS-31 significantly reduced IL1B protein (Fig. 5D). Because CH223191 is an AhR antagonist, we also measured CYP1B1, a canonical AhR-responsive detoxification gene. CYP1B1 expression at 24 hr was significantly downregulated by CH223191 indicate that it is indeed being inhibited (Fig. 5E). These results suggest that DMAKG, CH223191, and SS-31 may have therapeutic potential in mitigating the inflammatory response induced by DEP exposure.

**Figure 6:**
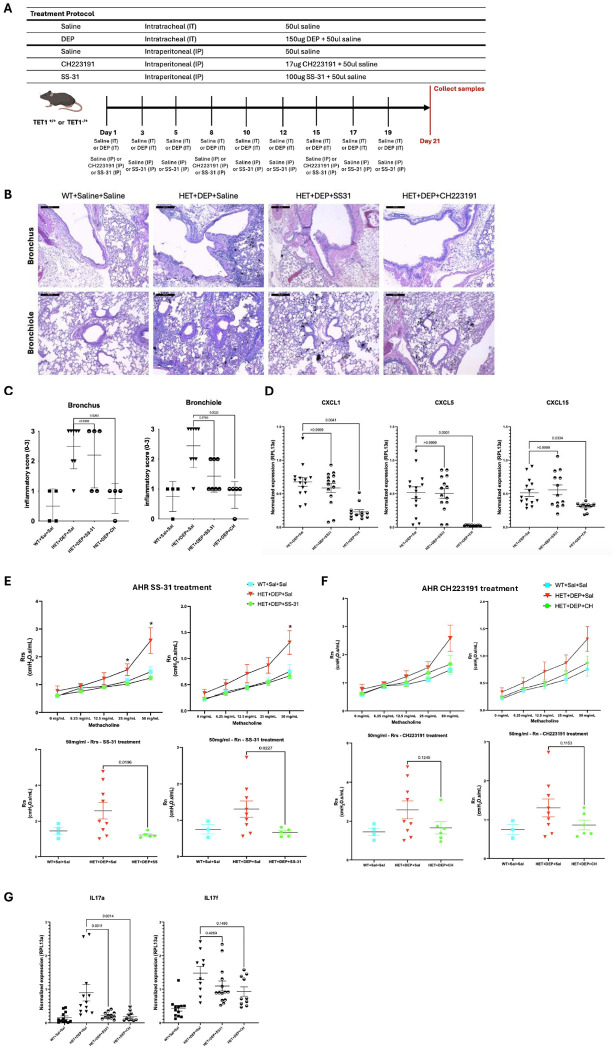
Effects of targeting TET1-regulated pathways *in vivo*. (A) Experimental design. TET1 heterozygous mutant (HET) and wild-type littermates (WT) mice were administered with intratracheal (IT) instillations of either saline or DEP (150 mg) 3 times per week for 3 weeks (9 IT instillations in total). Intraperitoneal (IP) injections of either saline, CH223191 (17mg), SS-31 (100mg) were given once a week (CH223191) or 3x per week (SS-31) at the same time as IT instillation. (B) Lung histology (H&E staining) of the bronchus (top) and bronchiole (bottom). A representative of all mice in the experiment is shown. (C) Quantification of lung inflammation. (D) Relative mRNA expression of proinflammatory cytokines (CXCL1, CXCL5, CXCL15) measured by qPCR. Welch’s t tests were applied. (E-F) Airway hyper-responsiveness (AHR) in murine model of DEP-induced lung inflammation and effect of treatment with SS-31 (E) or CH223191 (F). (top) The changes Rrs and Rn at all dosages of methacholine challenge test; (bottom) Rrs and Rn at 50mg/ml mch dose. Welch’s t tests were applied. *p<0.05. (G) Relative mRNA expression of proinflammatory cytokines (IL17a and IL17f) measured by qPCR. Welch’s t tests were applied. Animal numbers: WT+Sal, n=3–12; HET+DEP, n=8–14; HET+DEP(IT)+SS-31(IP), n=5–14; HET+DEP(IT)+CH223191(IP), n=4–12.

To determine whether the exaggerated DEP-induced inflammation and airway dysfunction in Tet1+/− mice could be therapeutically attenuated, we pharmacologically targeted either AhR signaling (CH223191) or mitochondrial oxidative stress (SS-31) during repeated DEP challenge. DMAKG was not tolerated in preliminary experiments with this model, so it was not further pursued *in vivo* (data not shown). Using the same 3-week intratracheal (IT) instillation model (saline or 150 μg DEP) applied across nine total instillations, we incorporated intraperitoneal (IP) dosing with either SS-31 (100 μg/IP, 9 IP in total) or CH223191 (17 μg/IP, 3 IP in total) according to the schedule shown in Fig. 6A. Lung histology of the bronchus and bronchiole (Fig. 6B), together with quantitative inflammation scoring (Fig. 6C), indicated that CH223191 produced the clearest reduction in DEP-associated airway inflammation relative to HET + DEP+Saline. Consistent with reduced airway inflammation, CH223191 also decreased lung expression of neutrophil recruitment-associated chemokines (CXCL1, CXCL5, and CXCL15; Fig. 6D). Functionally, methacholine challenge testing showed that SS-31 significantly reduced AHR (Rrs and Rn at 50mg/ml) in HET + DEP mice (Fig. 6E), whereas CH223191 produced only partial improvement (Fig. 6F). Consistent with our earlier findings that DEP-exposed HET mice exhibit elevated Th17-associated cytokines (Fig. 4A), Il17a and Il17f remained increased in HET + DEP+Saline compared with WT+Saline+Saline, and both SS-31 and CH223191 significantly reduced Il17a (Fig. 6G), which may contribute to the improved airway physiology observed in this model.

Collectively, these findings demonstrate that inhibiting AhR signaling and oxidative stress pathways downstream of TET1 effectively mitigates the heightened inflammatory response and impaired lung function caused by TET1 loss. Together, these results further support the role of TET1 as an upstream, central regulator that coordinates multiple pathways driving DEP-induced inflammation.

## Discussion

Environmental exposures, including traffic-related air pollution, are known to increase the risk of asthma susceptibility and severity via epigenetic regulation^3,9^. This study demonstrates a critical role for TET1 in modulating airway epithelial and lung responses to DEP, both at the transcriptomic and functional levels. Through an integrative approach employing TET1 knockdown in human bronchial epithelial cells (HBECs) and Tet1-deficient mice, we reveal that TET1 loss drives transcriptional changes, amplifies cellular and molecular responses to environmental pollutants, and heightens lung inflammation and airway hyper-responsiveness *in vivo*. We further explored whether selected TET1-linked pathways could be experimentally modulated using TET enzyme substrate DMAKG, the AhR antagonist CH223191, and the mitochondria-targeted peptide SS-31 *in vitro* and/or *in vivo*.

### TET1 alters baseline transcription of inflammatory markers and reshapes airway epithelial immune responses to subsequent stimuli with DEP.

Because TET1 expression itself is dynamic and altered by DEP in a dose- and time-dependent manner, it can be difficult to disentangle whether TET1 changes are a cause or consequence of inflammatory signaling during DEP exposure. Our TET1 knockdown and DEP-induced inflammation models allows us to isolate the transcriptional consequences of TET1 loss (TET1 KD group) both at baseline and in the context of DEP challenge (KD + DEP group). Our RNA-seq analysis revealed substantial overlap in the genes and pathways affected by both TET1 knockdown and DEP exposure, with a notable subset of genes uniquely regulated by each condition (see Fig. 1B). The compounded transcriptomic response observed after combined TET1 knockdown and DEP exposure (KD + DEP group) suggests that loss of TET1 may prime epithelial transcriptional networks in a way that alters subsequent pollutant responses. This is consistent with prior work showing that TET1 regulates airway epithelial inflammatory programs through modifying chromatin accessibility, DNA methylation and histone modifications, and can modulate AhR-related gene expression in HBECs under baseline and stimulated conditions^9^. Gene-level analysis of cytokine signaling indicated that TET1 is an important modulator of pro-inflammatory alarmins (IL-33, HMGB1) and cytokines (IL-1β, IL-8), both in non-exposed and DEP-exposed cells. These findings suggest that loss of TET1 reduces the repression of key inflammatory mediators, resulting in an exaggerated response to DEP. Whether TET1 plays similar roles in modulating responses to particulate matter from traffic and other sources will be examined in future studies.

### Correlative analyses link TET1-dependent molecular changes and neutrophilic inflammation to altered airway functional responses.

In mice, Tet1 deficiency increased DEP-induced airway hyperresponsiveness and lung inflammation, with prominent neutrophilic inflammation in BALF (Fig. 3). One mechanistic explanation for the association between IL-17A and airway hyperresponsiveness is that IL-17A may act directly on airway smooth muscle to enhance contractility, as previously proposed^28^. This interpretation is consistent with our observed relationship between Il17a expression and airway mechanics in DEP-exposed mice (Fig. 4D and **Supplemental Table 1**). Additionally, the correlations linking neutrophil concentrations to IL-17–associated signals and to CXCL5, as well as the association between BALF CXCL5 and airway resistance (Rrs) (Fig. 4C and D), support a mechanistic bridge from inflammatory programming to functional airway outcomes. This fits with published evidence that DEP can upregulate IL-17A in airway contexts and that DEP exposure can drive CXCL5-associated neutrophil recruitment *in vivo*^10^, reinforcing the plausibility of an IL-17/CXCL5 neutrophilic endotype contributing to the AHR phenotype observed in our study. It is important to note, however, that airway hyperresponsiveness is a multifactorial outcome, influenced not only by inflammation but also by structural remodeling, epithelial barrier dysfunction, smooth muscle hypercontractility, and altered neural regulation. Future studies will be needed to fully delineate the relative contributions of inflammatory versus structural and remodeling processes to the AHR phenotype observed in TET1-deficient animals exposed to DEP.

### TET1 maintains a balance between AhR-mediated detoxification and inflammatory signaling in response to DEP.

The AhR signaling pathway, which is critical for xenobiotic metabolism and immune modulation, was also significantly disrupted by TET1 loss. AhR is a ligand-activated transcription factor that regulates genes involved in xenobiotic metabolism and immune responses in a cell type specific manner^29^. Specifically, the role of AhR in asthma and inflammation is cell type and context dependent, which may explain some of the contradicting data we observed. For example, we observed a seeming mismatch between reduced AHR transcript levels and increased CYP1A1/CYP1B1 after DEP exposure (Fig. 2C). This may have occurred because CYP induction primarily reflects ligand-driven AhR activity through pre-existing receptor protein, while AHR mRNA is often shaped by time-dependent feedback and receptor turnover after activation^30^. In particular, AhR activation engages negative-feedback programs and can also trigger ligand-dependent AhR downregulation via proteasome-linked mechanisms^31,32^, which may explain why our single 24-h snapshot captured persistent CYP transcripts alongside lower AHR expression.

Additionally, in the KD + DEP vs DEP comparison, the concurrent reduction in canonical pathway components required for AhR transcriptional output, most notably ARNT, offers a potential explanation for why canonical detoxification targets such as CYP1A1 and CYP1B1 (and related ALDH genes) can drop even if DEP ligands are present [28]. At the same time, lower AHR/CYP transcript abundance does not necessarily indicate absence of detrimental AhR signaling, because AhR can influence inflammatory responses through context-dependent, non-canonical crosstalk with other signaling pathways such as NF-κB^33^; thus, blocking ligand binding can still dampen pro-inflammatory outputs even when canonical detox genes are blunted. In our model, TET1 loss was associated with downregulation of AhR gene expression and several detoxification enzymes (CYP1A1, CYP1B1, ALDHs), and upregulation of inflammatory cytokines and GSTP1 and HMOX1, additional detoxifying enzymes. This dichotomy supports the idea that TET1 helps maintain balance between AhR’s roles in environmental detoxification and inflammatory restraint, potentially by influencing both canonical and non-canonical signaling outputs. This conceptual framework, along with prior reports^33,34^ that AhR antagonism (including CH223191) can reduce pollutant- or allergen-exacerbated inflammatory responses *in vitro and in vivo*^35^, motivated our use of CH223191 in our study. Consistent with this rationale, CH223191 reduced inflammatory mediators *in vitro* and decreased *CYP1B1* expression (supporting pathway inhibition) (Fig. 5E), and it also reduced airway inflammation and chemokine expression *in vivo*, even though improvements in airway hyperresponsiveness were only partial (Fig. 6). These results are consistent with the discussed dual role of AhR signaling in lung inflammation and suggest the involvement of other TET1-regulated pathways in airway function.

### TET1 modulates NRF2-driven antioxidant defenses, which in turn shapes mitochondrial ROS levels and the severity of DEP-induced airway dysfunction and inflammation.

Oxidative stress emerged as a central factor in our DEP–TET1 model, consistent with the overrepresentation of NRF2-mediated oxidative stress response pathways in the transcriptomic analyses and the altered expression of key antioxidant/detoxification genes. In HBECs, TET1 deficiency was associated with altered expression of redox-defense genes, including reduction in SOD2 (mitochondrial MnSOD) and dysregulation of other antioxidant enzymes (e.g., NQO1, ALDHs) (Fig. 2C and 2E), suggesting impaired capacity to buffer oxidant burden during DEP exposure. HMOX1 is also a key protective detoxification enzyme and a widely used biomarker of oxidative stress/ROS and Nrf2 pathway activation^36,37^. Notably, HMOX1 was increased in TET1 KD HBECs during the acute 24-hour exposure window (Fig. 2E), whereas in the mouse KO + DEP versus WT + DEP comparison after the repeated exposure model, Hmox1 was downregulated (**Supplemental Fig. 2**), which may indicate that HMOX1 responds in a different manner during chronic versus acute exposures. Importantly, mitochondrial superoxide increased with TET1 knockdown (Fig. 2F), indicating that the mitochondrial stress is a meaningful source of oxidative stress in this context; because mitochondrial ROS can contribute to total ROS effects by amplifying redox-sensitive inflammatory pathways and damaging cellular macromolecules^38^, these mitochondrial changes provide a plausible mechanistic link between TET1 loss, heightened inflammatory transcription, and downstream dysfunction. The *in vivo* NRF2 gene-expression signatures (e.g., Hmox1, Sod2, GST-family members) further support a role for TET1 in reshaping of antioxidant responses following DEP challenge.

Therapeutically, these observations motivated targeting mitochondrial oxidative stress with SS-31, a mitochondria-targeted peptide reported to suppress airway inflammation and oxidative stress in airway disease models^39^. SS-31 improves mitochondrial function by binding to cardiolipin in the inner mitochondrial membrane, stabilizing electron transport chain organization, enhancing ATP production, and reducing ROS generation at their source^40,41^, and it distinguishes itself from a general antioxidant as it acts upstream by preserving mitochondrial structure and electron flow rather than simply scavenging ROS after they are produced. In our study, SS-31 treatment during repeated DEP exposure improved methacholine-induced airway hyperresponsiveness (Rrs and Rn) and reduced Il17a expression, which is notable due to connection between Il17a and AHR reported in other studies^10,28^, and was consistent with our data in DEP-only correlation analyses (Il17a vs Rn at 50 mg/ml methacholine) (Fig. 4E). However, SS-31 treatment did not improve the overall histology-based inflammation score and did not broadly reduce the other measured inflammatory chemokines/cytokines (including CXCL1, CXCL5, and CXCL15, IL17f). This supports the idea that mitochondrial oxidative stress imbalance during TET1 deficiency may contribute to DEP-associated airway dysfunction and may be acting separately from other inflammatory pathways. These findings highlight an important limitation that while SS-31 and CH223191 each attenuate select aspects of the TET1-deficiency phenotype, neither compound alone fully normalizes the inflammatory or functional endpoints. Future studies employing combinatorial targeting strategies, broader time-course analyses, and cell-type-specific models will be needed to fully resolve how TET1-regulated oxidative stress and detoxification programs interact to drive the DEP-induced airway dysfunction observed in this model.

Collectively, we propose that loss of TET1 dysregulates oxidative stress response pathways, including the AhR pathway, by downregulating many detoxifying enzyme genes that normally limit ROS and oxidative stress, thereby exacerbating cellular damage and promoting pro-inflammatory gene expression. Our data identify TET1 as a crucial upstream epigenetic regulator in balancing inflammatory responses and cellular detoxification mechanisms, particularly in the context of environmental exposures like DEP. By acting upstream of multiple pathways, TET1 may represent a more integrative therapeutic target than strategies focused on individual downstream pathways.

## Materials and methods

### HBEC growth and TET1 knockdown

HBECs (obtained from Dr. John Minna’s lab at UT Southwestern Medical Center^42^) were grown in 1X K-SFM supplemented with EGF, pituitary extract, and pen-strep (complete media). After cells reaching near confluency, cells were then incubated with 1X K-SFM with no serum supplements (minimal media) overnight. Transfection was performed using lipofectamine 3000 with 30 pmol/mL siRNA (ThermoFisher, control siRNA, AM4611; TET1 siRNA, assay ID: 147894) according to manufacturer’s protocol. Following 24hr transfection, saline or diesel exhaust particles (cDEP, provided by Ian Gilmour from EPA^43^) was added to HBEC at a concentration of 10 μg/cm^2^ (based on the well surface area) and incubated for additional 8 or 24 hours before collection. This DEP dose induced significant activation of *CYP1B1*, oxidative stress, and proinflammatory responses without apparent cell death and similar doses were used to examine the effects of real-life exposures^10,11,15,44–48^. Four different groups are included in our study: 1) Control (Control siRNA, no DEP), 2) DEP (with control siRNA), 3) KD (TET1 siRNA, no DEP), and 4) KD + DEP (TET1 siRNA, DEP). For testing the therapeutic molecules, following 24hrs of TET1 knockdown, Dimethyl 2-oxoglutarate (DMAKG, Sigma Aldrich, #349631–5G, final concentration 400 μM), CH223191 (Sigma Aldrich, #C8124, final concentration 10 μM), or SS-31 (novoprolabs, #318879, final concentration 100 nM) was added together with DEP.

### Murine model of DEP-induced lung inflammation

Tet1^+/−^ mice [heterozygous knockout/HET mice, Tet1tm1.1Jae/J (B6/129S4), Jackson lab Strain #:017358] and their littermate Tet1^+/+^ mice (wildtype/WT mice) were used. Tet1 deletion was confirmed via PCR. All animal procedures were approved by the Animal Experimental Ethics Committee of University of California, Davis. Mice (16 ~ 24g, age 8 ~ 12 weeks) were maintained under specific pathogen free conditions in temperature-controlled rooms with 12h dark and light cycles. The mouse model of DEP-induced lung inflammation was established as in previous studies^10,11^, which corresponds to cumulative exposures to an annual average DEP concentration of 20μg/m^3^ for 8 hours per day over a 5.7-months period in the general population, based on MPPD dosimetry model. In this model, 150μg of DEP (generated from a 4-cylinder Deutz diesel engine at the EPA, composition described by Dr. Ian Gilmour’s lab) or 50μl saline was administered via intratracheal (IT) instillations 3 times per week for 3 weeks (9 IT instillations in total). On day 21, airway hyper-responsiveness (AHR) was measured, bronchoalveolar lavage fluid (BALF) was collected, and lungs were harvested for pathology and DNA/RNA extraction. The same mouse model of DEP-induced lung inflammation was also used in examine the effects of therapeutic molecules, with additional intraperitoneal (IP) treatment added. IP injections of either saline, CH223191 (17μg), SS-31 (100μg) were given once a week (CH223191) or 3x per week (SS-31) at the same time as IT instillation.

### Measurement of mitochondria-specific ROS

HBEC were cultured, transfected and exposed as described above in a black-walled, clear bottom, and tissue-culture-treated 96-well plate (Corning, Glendale, AZ). Media was then removed and 100μL of 10 μM of MitoSOX red (MedChemExpress, Monmouth Junction, NJ) working solution was added to the wells and was incubated for 30 minutes in room temperature and in the dark. The plate was read using a microplate reader (Molecular Devices VERSAmax) at the excitation wavelength of 510 and the emission wavelength of 520.

### RNA-seq analysis and IPA

RNA-seq libraries were prepared for a total of 12 HBEC samples (Control n = 4; TET1KD n = 4; DEP n = 2; KD + DEP n = 2) and 11 mouse samples (WT-DEP n = 7; KO-DEP n = 4). RNA (0.5μg per sample, all with RNA quality ≥ 7) was submitted for poly-A RNA library preparation and sequencing (21–30 million PE150 reads/sample). Read quality was assessed using FastQC 3^49^. Reads were trimmed using Trim Galore [44] and aligned to the human transcriptome (hg38) using Bowtie2^50^. Transcripts were subsequently quantified using RSEM^51^. The data were then converted into DESeq2^52^ format using tximport^53^. DESeq2 was also used for differential expression analyses and for hierarchical clustering and principal component analyses. We used the “ashr” method in DESeq2 to shrink pairwise fold changes^54^. Genes with an absolute shrunken fold change of at least 1.2 and an FDR ≤ 0.05 were considered significantly differentially expressed (DE). Pathway analyses were performed using IPA (QIAGEN Inc., https://www.qiagenbioinformatics.com/products/ingenuitypathway-analysis) through Core Analysis. Significantly enriched Canonical Pathways were identified with a B-H Multiple Testing Correction p value ≤ 0.05 significance threshold. IPA activation z-scores quantify how strongly the observed up/down-regulation of pathway genes matches the direction of regulation expected from the IPA knowledge base, with positive scores indicating predicted activation and negative scores indicating predicted inhibition.

### Airway hyper-responsiveness (AHR)

Airway responsiveness was measured using FlexiVent apparatus (Scireq, Montreal, Canada), based on our previous study^9^. Briefly, mice were anesthetized with Ketamine (100mg/Kg) and Xylazine (10mg/Kg). Mouse tracheas were cannulated with a 20-gauge blunt needle, and the mice were ventilated at 150 breaths/min, 3.0 cmH2O positive end expiratory pressure (PEEP), and 10 mg/Kg tidal volume (Vt). Then, 5 different concentrations (0, 6.25, 12.5, 25, 50 mg/ml) of methacholine were used to challenge the airway.

### Bronchoalveolar lavage fluid (BALF) collection and analysis

BALF was collected according to our previous study^9^. Briefly, the BALF supernatant was isolated after centrifugation and stored at −80°C before cytokine assay. CXCL5, and CXCL15 in BALF were measured by ELISA (R&D Systems, USA). Meanwhile, total cell numbers were counted with a hemacytometer. Subsequently, cytospins were stained with the Hema 3 Staining System (Fisher Scientific, Kalamazoo, MI) and differential cell counts were determined.

### Hematoxylin and eosin (H&E) staining and quantification of Inflammatory Score

The left lung of each mouse was infused and fixed in 4% paraformaldehyde, embedded in paraffin, cut into 5μm sections, and stained with H&E to observe pulmonary pathological changes. A score from 0–3 was assigned to a bronchus and a proximal bronchiole of every sample. A proximal bronchiole is a bronchiole or a group of bronchioles that are located closest to the bronchus. The score is “0” if no inflammatory cells are detected, “1” if occasional inflammatory cells are present, “2” for moderate presence of the inflammatory cells, and “3” for heavy presence of inflammatory cells.

### RNA extraction and Quantitative PCR (qPCR)

Briefly, the frozen right upper lobe of each mouse was homogenized with ceramic beads (Omni Inc, Kennesaw, Georgia) and the Omni Bead Ruptor 24 (Omni Inc, Kennesaw Georgia), or Qiasshredder (Qiagen, Valencia, CA). Total RNA was isolated from homogenized lung tissues either by TRIzol (Invitrogen, Carlsbad, CA) extraction followed by cleanup with the RNeasy MinElute Cleanup Kit, or directly using the RNeasy Mini Kit (Qiagen, Valencia, CA), according to the manufacturers’ instructions. RNA from HBECs was extracted using the RNeasy Mini Kit (Qiagen, Valencia, CA) according to manufacturer’s instructions. cDNA was made using SuperScript VILO IV Master Mix (ThermoFisher ID: 11756050). Quantitative PCR was performed with PowerSYBR Green PCR Master Mix (Applied biosystems, Woolston, Warrington, UK) as a ready-to-use reaction mixture on QuantStudio 12k Flex qPCR machine. RPLP0 was used as an internal control for HBECs and Rpl13a was used for mouse experiments.

### Statistical analysis

All statistical analyses were performed using GraphPad Prism 9.0. All data were presented as mean ± standard error of the mean (SEM). Welch’s t tests were applied for two-group comparisons, which do not assume equal variances. One-way ANOVA with Tukey's post hoc or Šídák's multiple comparisons tests were applied when more than two groups were compared. The outliners identified by Grubbs test were excluded. The Spearman rank correlation coefficient was calculated to analyze correlations. A threshold of p < 0.05 was considered statistically significant.

## Supplementary Material

Supplementary Files

This is a list of supplementary files associated with this preprint. Click to download.

- SupplementaryInformation20260519.pdf
- SupplementalFigure1v2.pdf
- SupplementalFigure2v2.pdf
- SupplementaryTable1.DEPHETmicecorrelationsresults.pdf

## Data Availability

RNA-seq data will be deposited to GEO upon manuscript acceptance.
